# Supplementary material for: Single-cell analysis reveals region-heterogeneous responses in rhesus monkey spinal cord with complete injury
Source: Nat Commun. 2023 Aug 9;14:4796. doi: 10.1038/s41467-023-40513-5 (PMC10412553; doi:10.1038/s41467-023-40513-5)
Supplement: Supplementary file 1 — Supplementary Information [file 41467_2023_40513_MOESM1_ESM.pdf]

Supplementary Materials for

Single-cell analysis reveals region-heterogeneous responses in rhesus monkey spinal cord with complete injury

Yongheng Fan *et al.*

Corresponding authors: Zhifeng Xiao, zfxiao@genetics.ac.cn; Yannan Zhao, ynzhao@genetics.ac.cn; Jianwu Dai, jwdai@genetics.ac.cn.

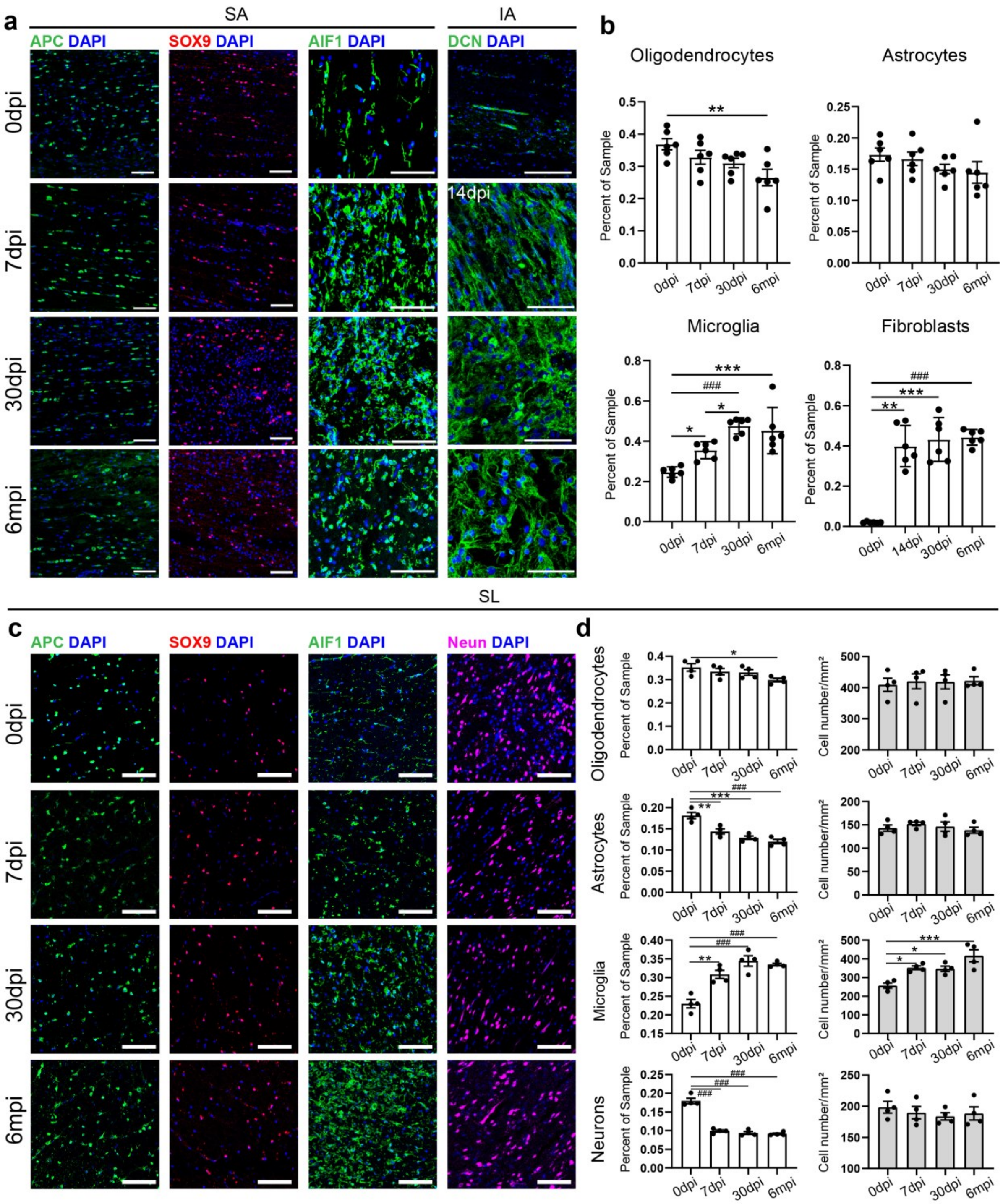

Supplementary Fig. 1 Immunostaining of dynamic changes of cells proximal and distal to the lesion after complete spinal cord injury. a Immunostaining images showing the distribution of oligodendrocytes (APC), astrocytes (SOX9), microglia (AIF1) in SA, and fibroblasts in IA of the completely injured rhesus monkey spinal cord at 0 dpi, 7 dpi, 30 dpi, and 6 mpi. Scale bars,

100 μm. **b** Quantification of the proportion of cell types from the immunostaining in the tissue of SA and IA at 0 dpi, 7 dpi, 30 dpi, and 6 mpi. Data are shown as mean ± SEM, n= 6 slices. *p < 0.05, **p < 0.01, ***p < 0.001, ###p < 0.0001, one-way ANOVA coupled with Tukey's post hoc test. **c** Immunostaining images showing the distribution of oligodendrocytes (APC), astrocytes (SOX9), and microglia (AIF1) in SL of the completely injured rhesus monkey spinal cord at 0 dpi, 7dpi, 30dpi, and 6 mpi. Scale bars, 100 μm. **d** Quantification of the proportion (left) and number (right) of cell types from the immunostaining in the lumbar tissue at 0dpi, 7dpi, 30dpi, and 6mpi. Mean ± SEM, n= 4 slices. *p < 0.05, **p < 0.01, ***p < 0.001, ###p < 0.0001, one-way ANOVA coupled with Tukey's post hoc test. Source data are provided as a Source Data file.

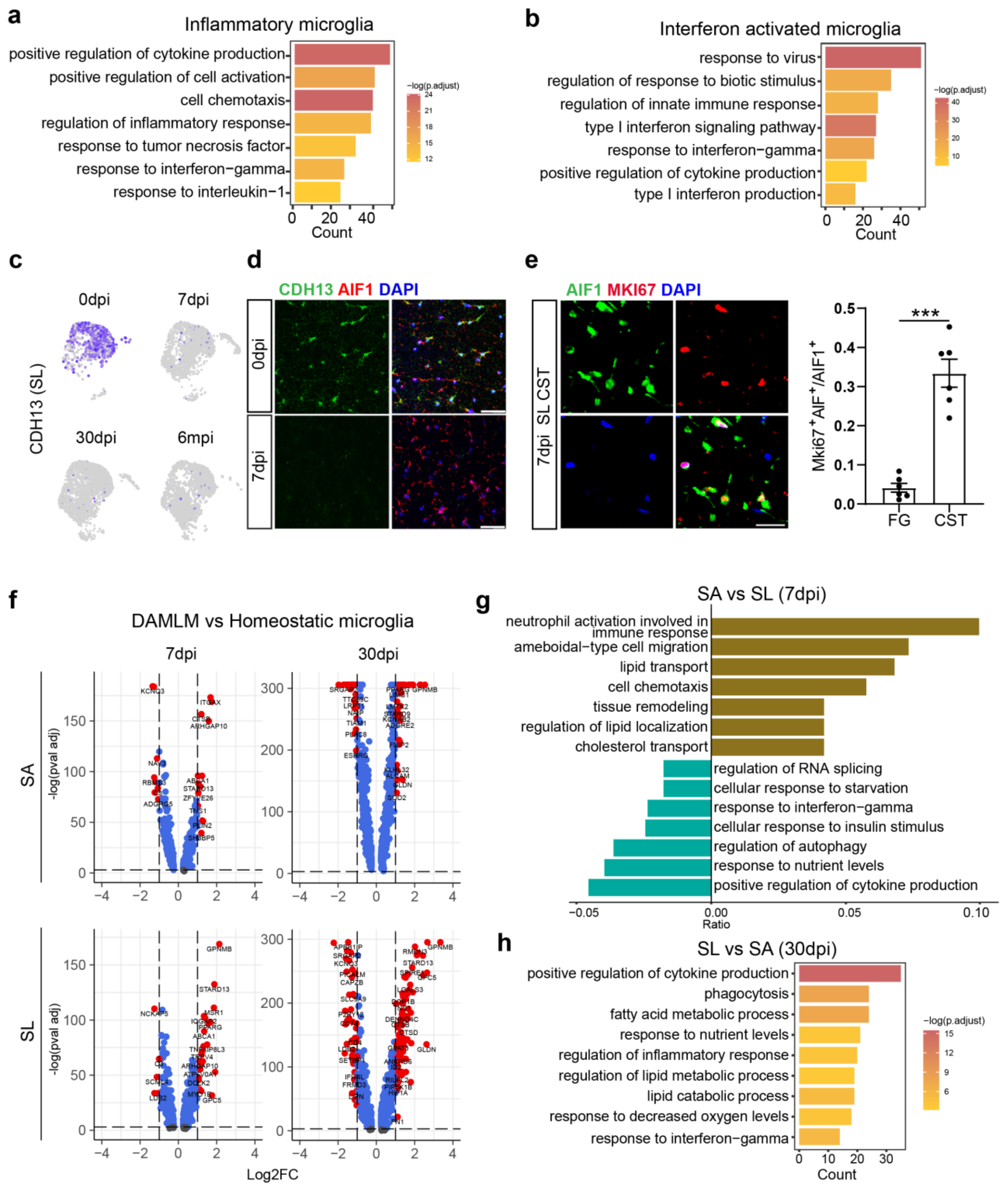

Supplementary Fig. 2 Microglia are heterogeneously activated in both proximal and distal regions after SCI. a-b Enriched GO biological process terms of the highly expressed genes in inflammatory microglia and interferon-activated microglia. *P* values (adjusted) were calculated using Benjamini–Hochberg false discovery rate (FDR). **c** Expression of *CDH13* in microglia of

the spared lumbar at different time points after SCI. **d** Immunostaining of AIF1 and CDH13 in the lumbar sections of uninjured and injured rhesus monkey spinal cord. Scale bars, 25 μm. **e** Immunostaining of AIF1 and MKI67 in the lumbar sections of completely injured rhesus monkey spinal cord at 7dpi (left) and quantification of the proportion (right) of MKI67 positive microglia in CST and FG regions of the lumbar tissue at 7dpi. Scale bars, 50 μm. Mean ± SEM, n= 6 slices. ***$p < 0.0001$, two-sided Student's *t*-test. CST, corticospinal tract; FG, fasciculus gracilis. **f** Volcano plots showing differentially expressed genes (DEGs) of DAMLM compared with homeostatic microglia in SA and SL. *P* values (adjusted) were calculated using Wilcoxon Rank Sum test followed by Bonferroni correction. **g** Enriched GO biological process terms for the DEGs of DAMLM in SA (right) compared with that in SL (left) at 7 dpi. The ratio represents the number of genes enriched on the GO term among the total number of genes that were input for enrichment analysis. **h** Enriched GO biological process terms for the upregulated genes of DAMLM in SL compared with that in SA at 30 dpi. *P* values (adjusted) were calculated using Benjamini–Hochberg false discovery rate (FDR). Source data are provided as a Source Data file.

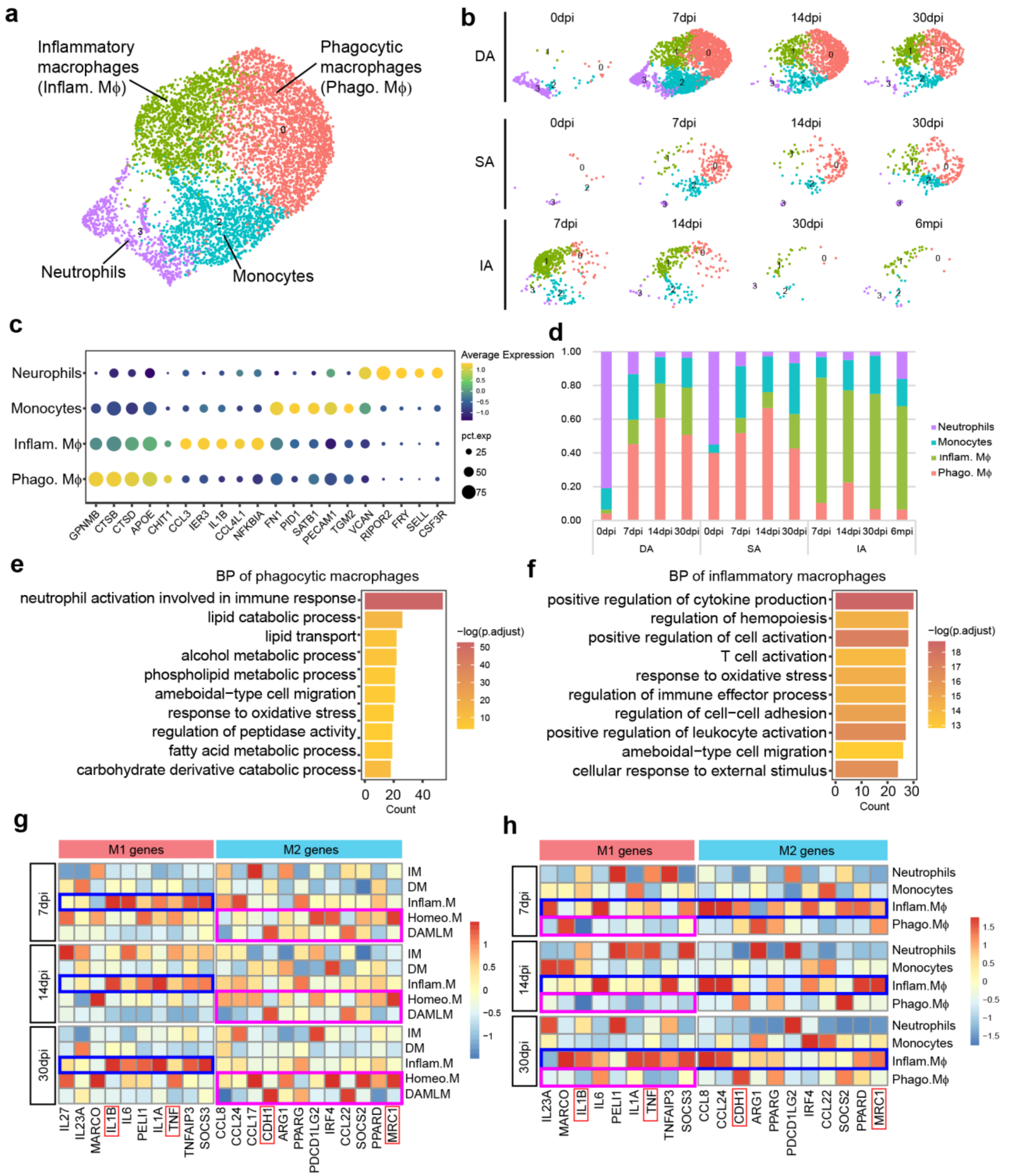

Supplementary Fig. 3 Inflammatory characteristics of inflammatory cell subsets after SCI. **a** Heterogeneous subsets of inflammatory cells. **b** Split UMAP plots depicting the regional distribution of inflammatory cells at distinct time points after SCI. **c** Differentially expressed genes

among subsets of inflammatory cells. The size of the dot indicates the percentage of cells in which that gene is detected and the color bar is scaled with the average expression of the corresponding genes. **d** Bar plot showing the dynamic proportion of inflammatory cells in different regions. **e-f** Enriched GO terms for highly expressed genes of phagocytic macrophages and inflammatory macrophages. *P* values (adjusted) were calculated using Benjamini–Hochberg false discovery rate (FDR). **g-h** Heatmaps showing the expression of M1 and M2 macrophage genes in subsets of inflammatory cells. The color bar is scaled with the average expression of the corresponding genes. Source data are provided as a Source Data file.

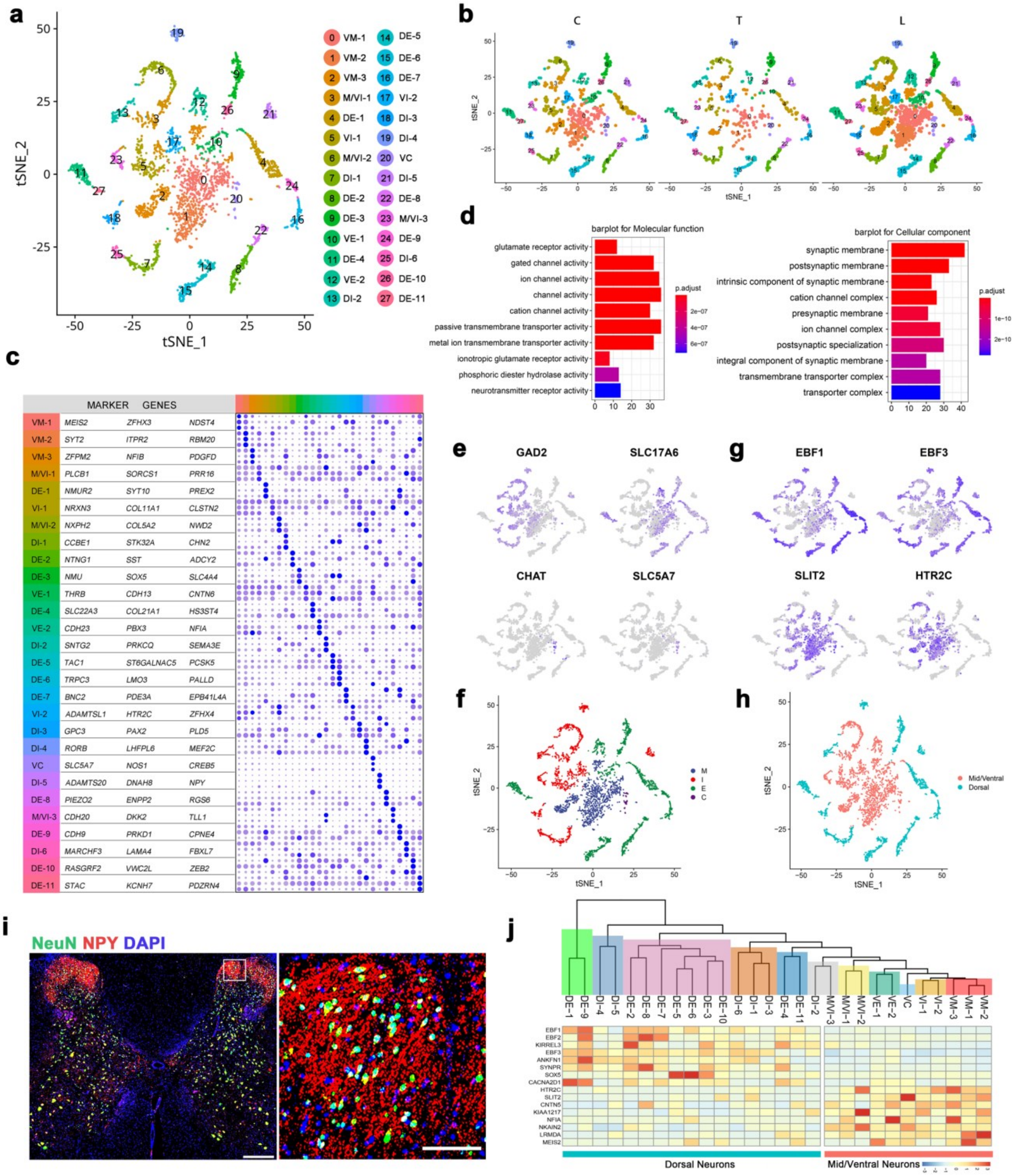

Supplementary Fig. 4 Neuron populations show heterogeneity related to neurotransmitter status and spatial location. a t-SNE plot showing the heterogeneous subsets of neurons in the uninjured spinal cord. Clusters are labeled according to spatial location and transmitter status. D,

dorsal; M/V, middle/ventral; V, ventral; E, excitatory; I, inhibitory; M, mixed; C, cholinergic. **b** Split t-SNE plots show the distribution of neuronal subsets in different segments. C, cervical; T, thoracic; L, lumbar. **c** Representative signature genes of each neuronal subtype. The size of the dot indicates the percentage of cells in which that marker is detected and the color bar is scaled with the average expression of the corresponding genes. **d** GO enrichment analysis of the top 20 DEGs of all neuronal subsets. *P* values (adjusted) were calculated using Benjamini–Hochberg false discovery rate (FDR). **e-f** Gene expression visualized by t-SNE plot depicting the neurotransmitter characteristic of neuronal subsets. **g-h** Gene expression visualized by t-SNE plot depicting the spatial location characteristic of neuronal subsets. **i** Immunostaining showing the distribution of NPY-expression neurons in rhesus monkey spinal cord. The areas in white boxes are shown at high magnification. scale bar, 500 μm in the left and 50 μm in the right. (**j**) Hierarchical clustering of the neuronal subsets. A lower distance indicates more similar gene expression between neuronal subsets. Heatmap showing DEGs between dorsal and ventral neuronal subtypes. The color bar is scaled with the average expression of the corresponding genes.

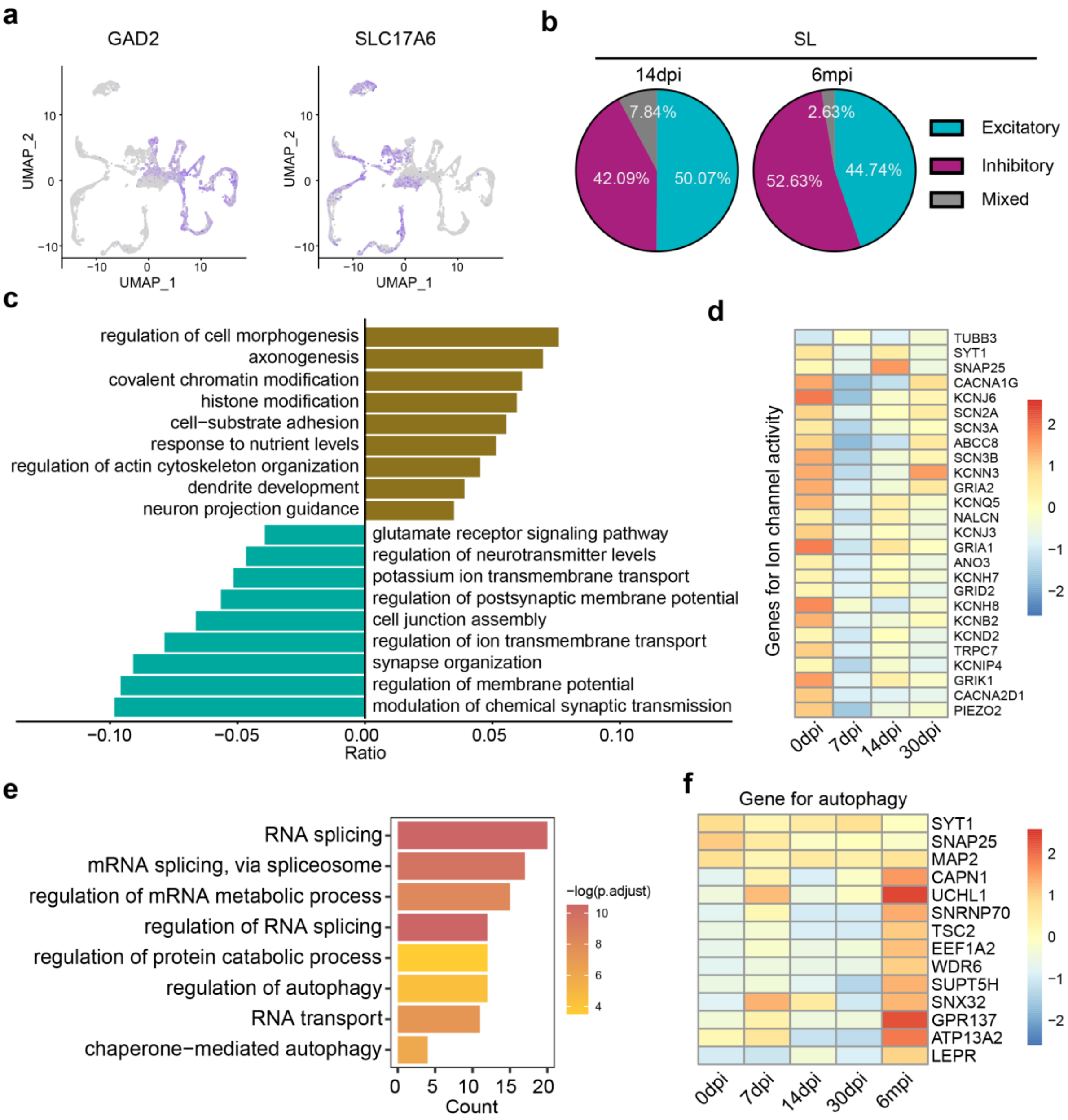

Supplementary Fig. 5 The spatiotemporal heterogeneous responses of neurons after SCI. a Expression of *GAD2* (inhibitory neuron) and SLC*17A6* (excitatory neuron) in neuronal subsets from the intact and injured spinal cord. **b** Pie plots showing the proportion of neurons with different neurotransmitters in the distal lumbar at 14 dpi and 6 mpi. **c** Enriched GO terms for the upregulated and downregulated genes of neurons in the SA area at 7 dpi compared with that in the uninjured spinal cord. The ratio represents the number of genes enriched on the GO term among the total

number of genes that were input for enrichment analysis. **d** Heatmap showing the expression of genes related to ion channel activity in neurons from the SA area. *SYT1*, *TUBB3*, and *SNAP25* were used as marker genes to indicate neuron identity. The color bar is scaled with the average expression of the corresponding genes. **e** Enriched GO terms for the upregulated genes in neurons from the SL area 6 months after SCI. *P* values (adjusted) were calculated using Benjamini–Hochberg false discovery rate (FDR). **f** Heatmap showing expression of genes related to autophagy in the neuronal population from the SL area. *SYT1*, *MAP2*, and *SNAP25* were used as marker genes to indicate neuron identity. The color bar is scaled with the average expression of the corresponding genes. Source data are provided as a Source Data file.

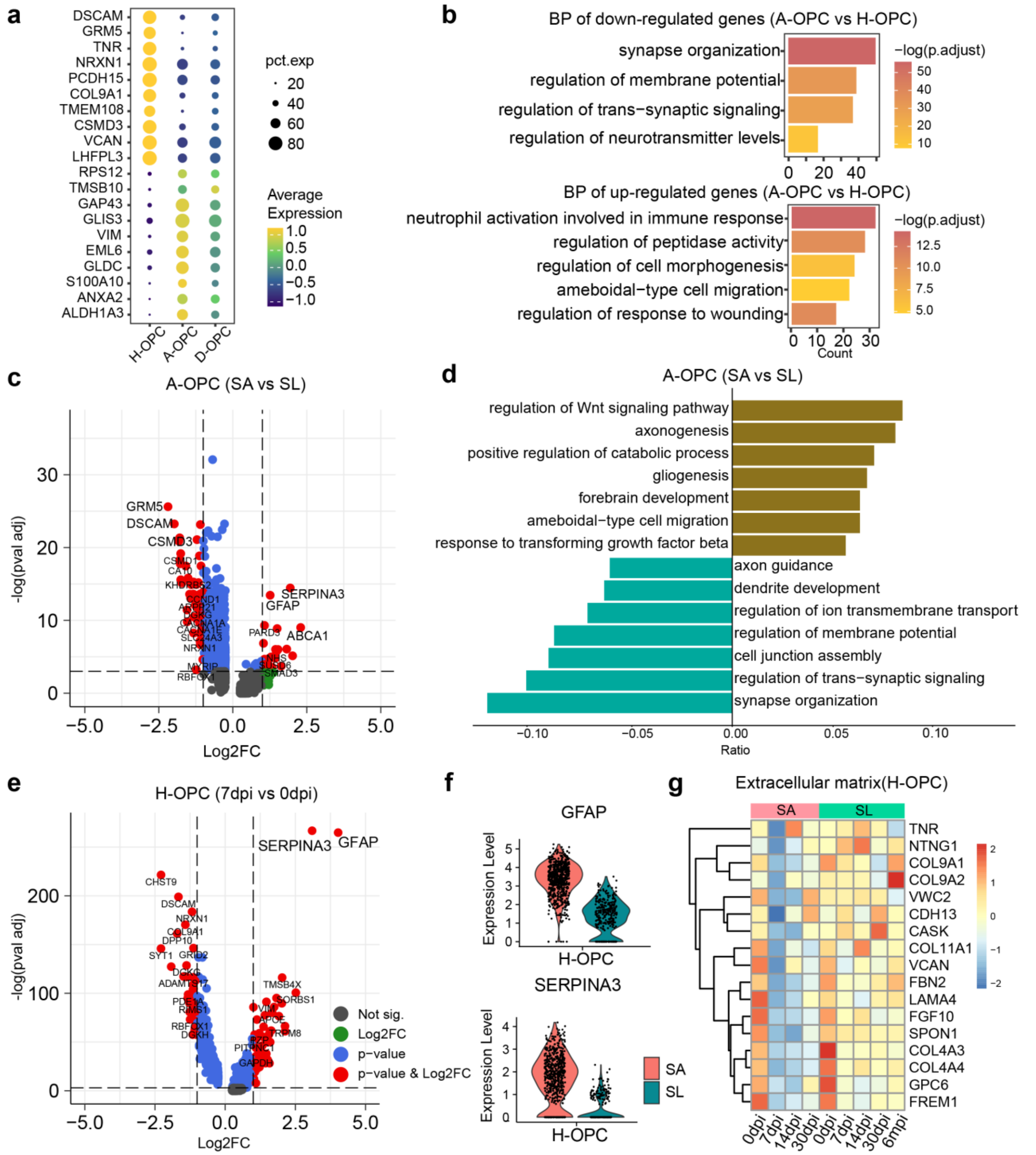

Supplementary Fig. 6 Oligodendrocyte progenitor cells (OPCs) are persistently activated in proximal regions but are slightly activated in distal regions after SCI. a Differentially expressed genes between A-OPC and H-OPC. The size of the dot indicates the percentage of cells in which that gene is detected and the color bar is scaled with the average expression of the

corresponding genes. **b** GO enrichment analysis of the upregulated genes and downregulated genes of A-OPC compared with H-OPC. *P* values (adjusted) were calculated using Benjamini–Hochberg false discovery rate (FDR). **c** Volcano plots show the differentially expressed genes of A-OPC in the SA compared to that in the SL area at 7 dpi. *P* values (adjusted) were calculated using Wilcoxon Rank Sum test followed by Bonferroni correction. **d** GO enrichment analysis of the upregulated genes and downregulated genes of A-OPC in the SA compared to that in the SL area at 7dpi. The ratio represents the number of genes enriched on the GO term among the total number of genes that were input for enrichment analysis. **e** Volcano plots showing differentially expressed genes of H-OPC at 7dpi compared with that at 0dpi. *P* values (adjusted) were calculated using Wilcoxon Rank Sum test followed by Bonferroni correction. **f** Violin plots depicting the expression levels of *GFAP* and *SERPINA3* in H-OPC from the SA and SL regions at 7 dpi. **g** Heatmap showing the expression of genes related to extracellular matrix organization in H-OPC from the SA and SL area after SCI. The color bar is scaled with the average expression of the corresponding genes.

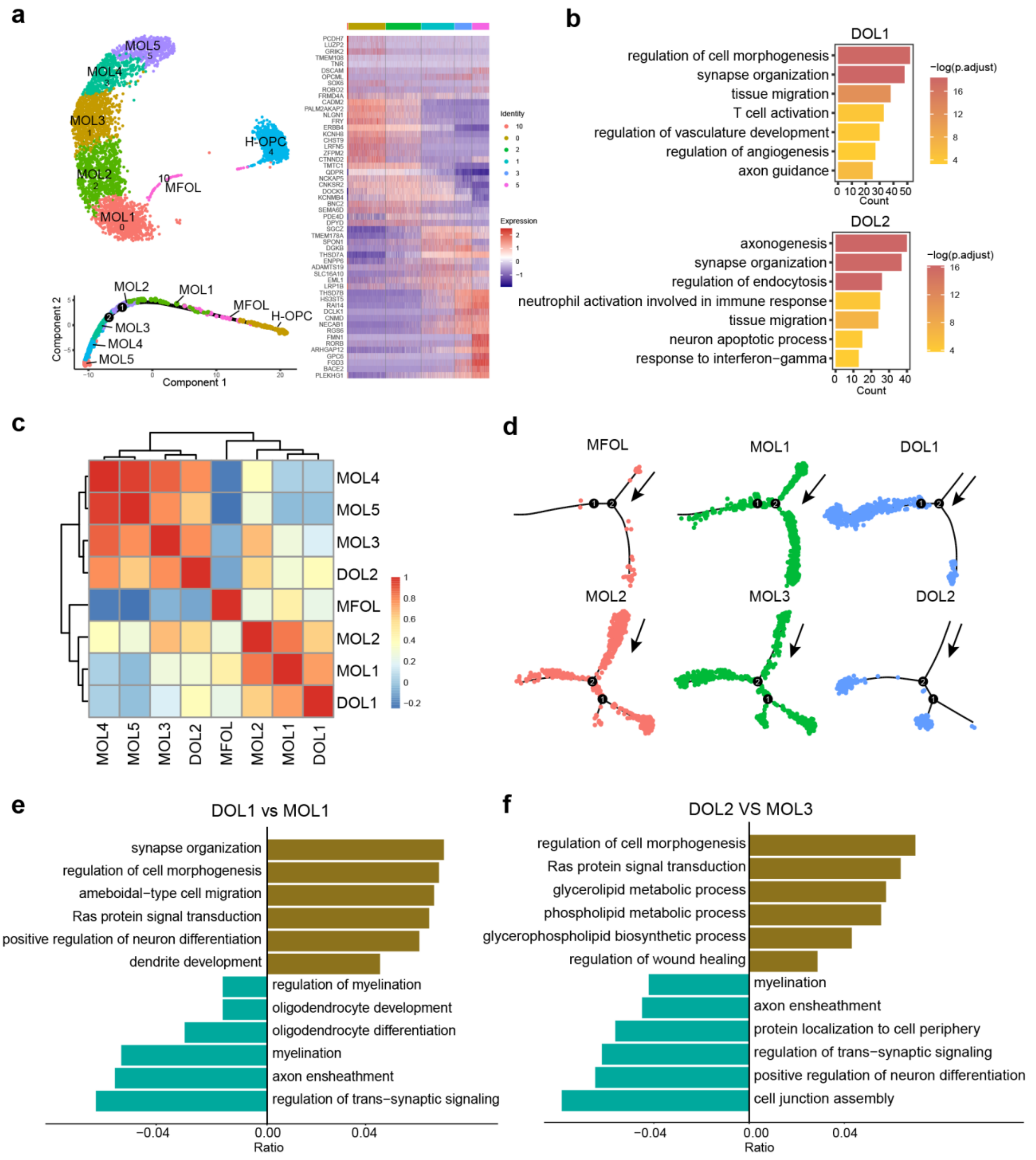

Supplementary Fig. 7 Characteristics of newly emerging disease-associated oligodendrocytes (DOLs) after SCI. a UMAP plot and pseudo-time analysis depicting the differentiation trajectory from OPCs to oligodendrocytes (left) and heatmap showing the gradual transition of DEGs among oligodendrocyte subsets (right). **b** Enriched GO terms for the highly expressed genes of DOL1 and

DOL2 compared with all other oligodendrocytes. *P* values (adjusted) were calculated using Benjamini–Hochberg false discovery rate (FDR). **c** Analysis of the similarity among oligodendrocytes subsets based on Pearson's correlation coefficient with top expression signature markers. The color bar indicates Pearson's correlation coefficient. **d** Pseudo-time analysis showing the trajectories from the preexisting oligodendrocyte subsets starting point to the DOLs branch. Colors represent different oligodendrocyte subsets. **e-f** GO enrichment analysis of the upregulated genes and downregulated genes of DOL1 compared with MOL1 and DOL2 compared with MOL3. The ratio represents the number of genes enriched on the GO term among the total number of genes that were input for enrichment analysis.

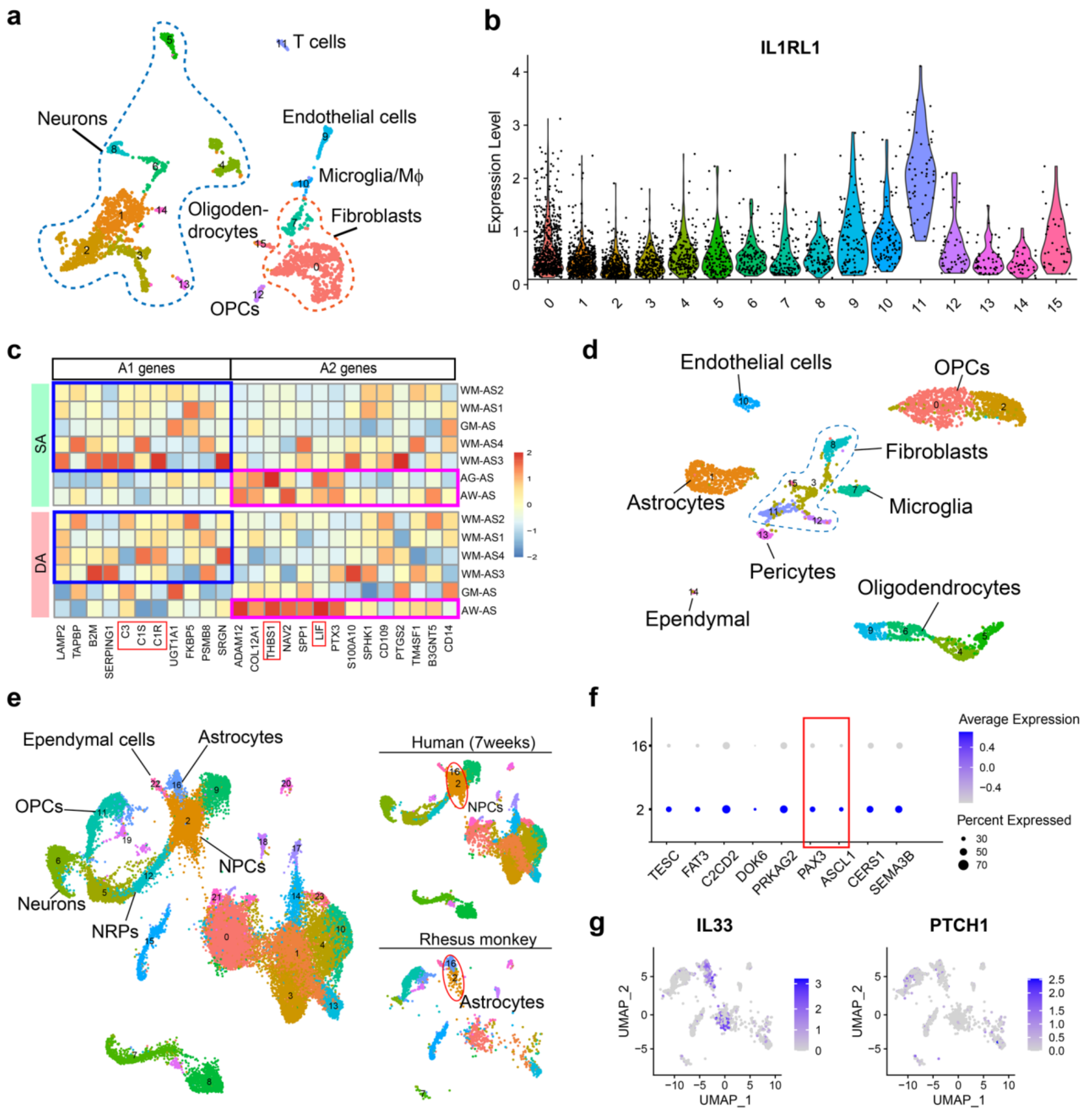

Supplementary Fig. 8 Different functions of white matter astrocytes and gray matter astrocytes. a Cluster analysis of cells expressing IL33 receptor gene *IL1RL1* in rhesus monkey injured spinal cord. **b** Violin plots showing the expression level of *IL1RL1* in each cluster of (a). **c** Heatmap showing expression of genes for A1 and A2 astrocyte phenotypes in astrocyte subsets from DA and SA after SCI. The color bar is scaled with the average expression of the corresponding genes. **d** Cluster analysis of cells expressing *NESTIN* in SA, DA, and IA after SCI.

e Integration of human embryonic spinal cord cells (7 weeks gestation) and *NESTIN*-positive cells of injured rhesus monkey spinal cord. Cells were annotated according to the well-known signature genes of spinal cord cells. **f** Dot plot showing the up-regulated genes of cluster 2 compared with cluster 16 in the lower right panel of (e). The size of the dot indicates the percentage of cells in which that marker was detected and the color bar is scaled with the average expression of the corresponding genes. **g** Expression of white matter (*IL33*) and gray matter astrocytes marker gene (*PTCH1*) visualized by UMAP.

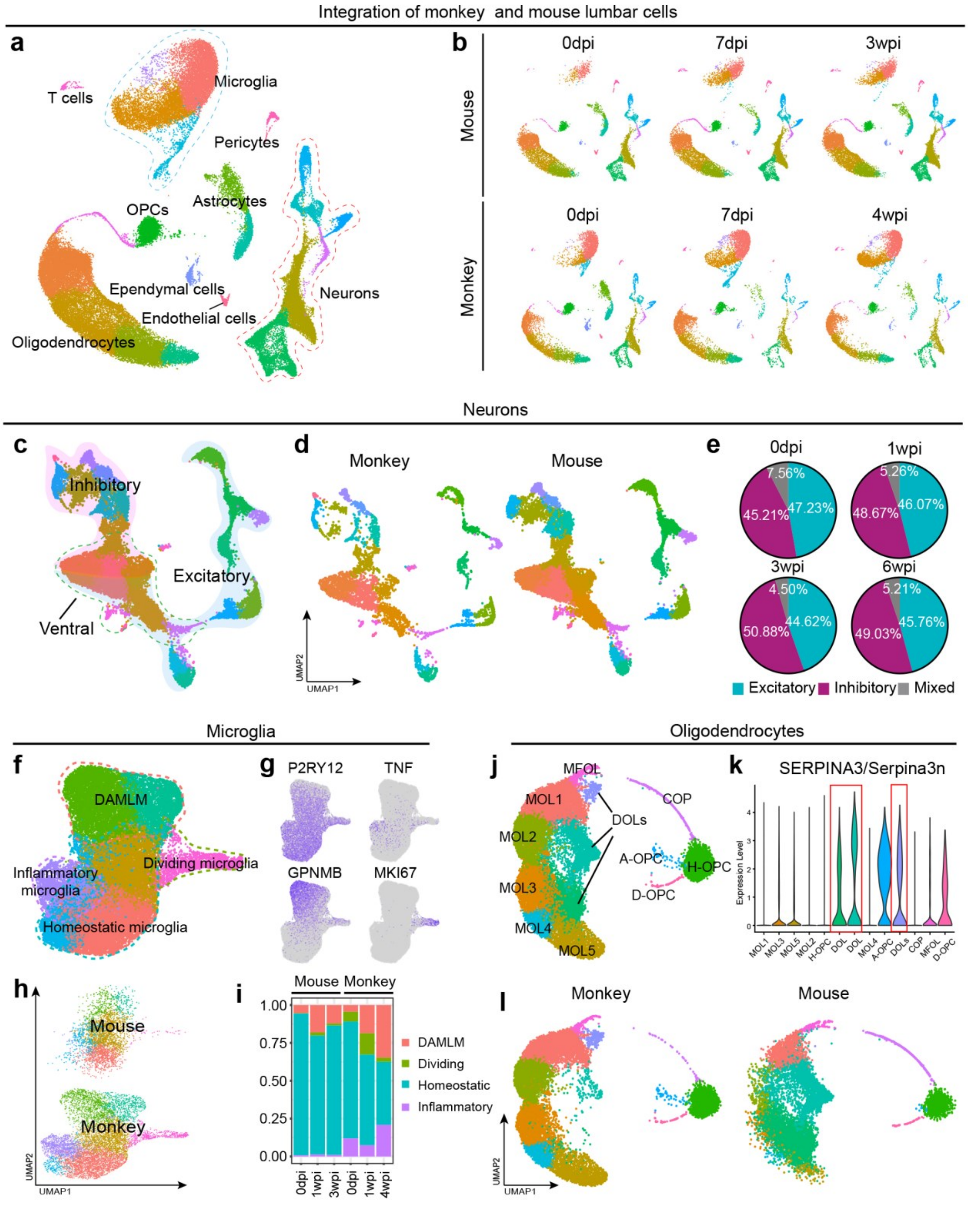

Supplementary Fig. 9 Conserved cellular responses in rhesus monkey and mouse lumbar after thoracic spinal cord injury. a Integration of rhesus monkey and mouse lumbar spinal cord cells. **b** Split UMAP plots showing the distribution of mouse and monkey lumbar cells at different

time points after SCI. **c-d** Integration of neurons from rhesus monkey and mouse lumbar spinal cord shows similar cluster patterns that are related to neurotransmitter status and spatial distribution. **e** Pie plots showing the proportion of neurons with different neurotransmitters in the mouse lumbar after SCI. **f** Integration of microglia from rhesus monkey and mouse lumbar spinal cord. **g** Gene expression visualized by UMAP plots. **h** Split UMAP plots showing the microglia distribution in mouse and monkey lumbar after SCI. **i** Cell percentage dynamic of microglia subsets in the rhesus monkey and mouse lumbar after SCI. **j** Integration of oligodendrocytes and OPCs from rhesus monkey and mouse lumbar spinal cord. **k** Violin plots showing the expression level of *SERPINA3/Serpina3n* in oligodendrocyte and OPC subsets from rhesus monkey and mouse lumbar spinal cord. **l** Split UMAP plots showing the distribution of oligodendrocyte and OPC subsets in mouse and monkey lumbar spinal cord after SCI. Source data are provided as a Source Data file.

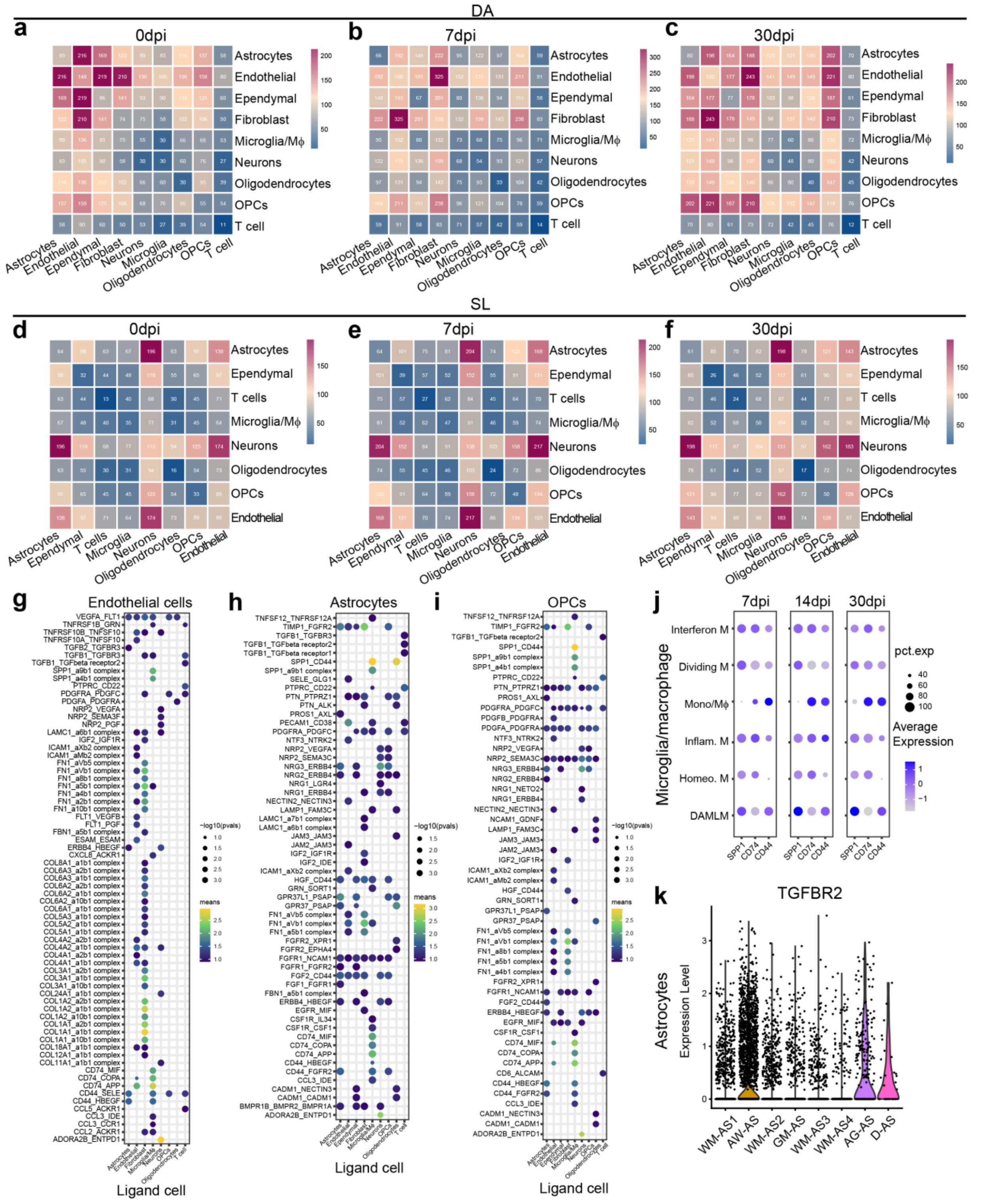

Supplementary Fig. 10 Ligand-receptor analysis to assess the cellular interaction in the proximal and distal regions after SCI. a-f Heat map depicting cell-cell communications between all identified cell types in the DA (a-c) and SL (d-f) derived from ligand-receptor interaction counts.

Interaction strengths are color coded with ligand-receptor counts. **g-i** Dot plot of the interaction scores between the ligand-expressing cells (bottom axis) and receptor-expressing endothelial cells (g), astrocytes (h), and OPCs (i) in the DA 7 days after SCI. Specific ligand-receptor pairs are listed along the left axis. *P* values are indicated by circle size. The color of the dot indicates the interaction score that bright yellow dots signify stronger interactions. *P* value is calculated on the basis of the proportion of the means that are as high as or higher than the actual mean. **j** Dot plots showing the expression of ligand gene in microglia and macrophage subsets in the DA at 7dpi. The size of the dot indicates the percentage of cells in which that marker was detected and the color bar is scaled with the average expression of the corresponding genes. **k** Violin plots showing the TGFB1 receptor gene *TGFBR2* expression level in astrocytes subsets in the DA at 7dpi.

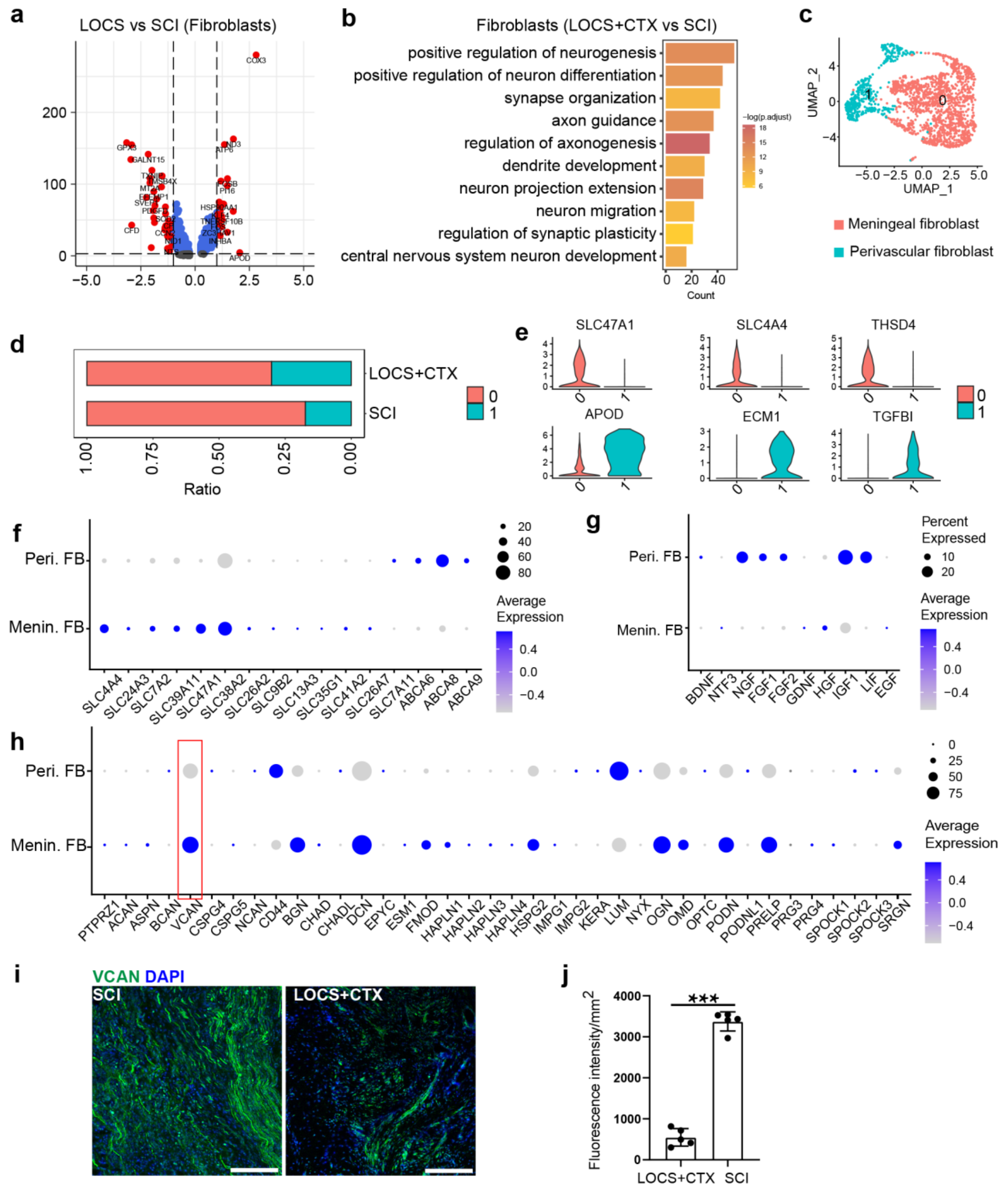

Supplementary Fig. 11 Functional scaffold implantation promotes the migration of perivascular fibroblasts into the injury area. a Volcano plots showing the differentially expressed genes of fibroblasts between the group with and without scaffold implantation. **b** GO

enrichment analysis of the upregulated genes in fibroblasts after scaffold implantation. *P* values (adjusted) were calculated using Benjamini–Hochberg false discovery rate (FDR). **c** UMAP plots depicting the fibroblast heterogeneity in the injury area after SCI with and without scaffold implantation at six months. **d** Bar plot showing the proportion of fibroblast subsets after SCI with and without scaffold implantation at six months. **e** Violin plots showing the signature genes of meningeal and perivascular fibroblasts. **f** Dot plot showing the expression of SLC and ABC family genes across perivascular (peri.) and meningeal (menin.) fibroblasts. The size of the dot indicates the percentage of cells in which that marker was detected and the color bar is scaled with the average expression of the corresponding genes. **g-h** Dot plot showing the expression of growth factors and proteoglycans genes across perivascular and meningeal fibroblasts. The size of the dot indicates the percentage of cells in which that marker was detected and the color bar is scaled with the average expression of the corresponding genes. **i** Immunostaining showed decreased deposition of VCAN in the injury area after scaffold implantation. Scale bar, 200 μm. **j** Quantitative analysis showing the mean fluorescence intensity of VCAN (per mm^2) in the injury area. Data are shown as mean ± SEM, n= 5 slices per group. ****p* < 0.0001, two-sided Student's *t*-test. Source data are provided as a Source Data file.
