# Supplementary material for: Single-cell analysis reveals region-heterogeneous responses in rhesus monkey spinal cord with complete injury
Source: Nat Commun. 2023 Aug 9;14:4796. doi: 10.1038/s41467-023-40513-5 (PMC10412553; doi:10.1038/s41467-023-40513-5)
Supplement: Supplementary file 3 — Reporting Summary [file 41467_2023_40513_MOESM3_ESM.pdf]

nature portfolio

Corresponding author(s): Zhifeng xiao, Yannan Zhao, Jianwu Dai

Last updated by author(s): Jul 26, 2023

Reporting Summary

Nature Portfolio wishes to improve the reproducibility of the work that we publish. This form provides structure for consistency and transparency in reporting. For further information on Nature Portfolio policies, see our Editorial Policies and the Editorial Policy Checklist.

Statistics

For all statistical analyses, confirm that the following items are present in the figure legend, table legend, main text, or Methods section.

n/a	Confirmed	
☐	☒	The exact sample size (*n*) for each experimental group/condition, given as a discrete number and unit of measurement
☐	☒	A statement on whether measurements were taken from distinct samples or whether the same sample was measured repeatedly
☐	☒	The statistical test(s) used AND whether they are one- or two-sided<br>*Only common tests should be described solely by name; describe more complex techniques in the Methods section.*
☒	☐	A description of all covariates tested
☐	☒	A description of any assumptions or corrections, such as tests of normality and adjustment for multiple comparisons
☐	☒	A full description of the statistical parameters including central tendency (e.g. means) or other basic estimates (e.g. regression coefficient) AND variation (e.g. standard deviation) or associated estimates of uncertainty (e.g. confidence intervals)
☐	☒	For null hypothesis testing, the test statistic (e.g. F, t, r) with confidence intervals, effect sizes, degrees of freedom and P value noted<br>*Give P values as exact values whenever suitable.*
☒	☐	For Bayesian analysis, information on the choice of priors and Markov chain Monte Carlo settings
☒	☐	For hierarchical and complex designs, identification of the appropriate level for tests and full reporting of outcomes
☐	☒	Estimates of effect sizes (e.g. Cohen's *d*, Pearson's *r*), indicating how they were calculated

Our web collection on statistics for biologists contains articles on many of the points above.

Software and code

Policy information about availability of computer code

Data collection	For scRNA-seq data, 10XGenomics and Cell Ranger v6.0.0 were used.<br>Images for immunohistochemistry, TUNEL assay and lipid droplet staining were acquired using the Leica SP8 confocal microscope set up (Leica).<br>Images for TEM assay were acquired using the HITACHI H-7650B transmission electron microscope.
Data analysis	Data analysis was performed using software Cellranger (6.0.0) and downstream analysis was done in R (v 4.0.5), using the following packages: DoubletFinder (v 2.0.3), Seurat (v 4.0.2), ggplot2 (v 3.3.6), ggrepel (v 0.9.1), plyr (v 1.8.7), dbplyr (v 2.1.1), tidyverse (v 1.3.1), monocle (v 2.3.6), ClusterProfiler (v 3.18.1), ggtree (v 2.4.2), CellphoneDB (v 4.0.0), EnhancedVolcano (v 1.8.0), and pheatmap (v 1.0.12).<br>Confocal images were processed with ImageJ (v 2.0.0) software.<br>Statistic analysis was performed by GraphPad Prism (v 8.0.2).

For manuscripts utilizing custom algorithms or software that are central to the research but not yet described in published literature, software must be made available to editors and reviewers. We strongly encourage code deposition in a community repository (e.g. GitHub). See the Nature Portfolio guidelines for submitting code & software for further information.

Data

Policy information about availability of data

All manuscripts must include a data availability statement. This statement should provide the following information, where applicable:

- Accession codes, unique identifiers, or web links for publicly available datasets
- A description of any restrictions on data availability
- For clinical datasets or third party data, please ensure that the statement adheres to our policy

The raw sequencing data and processed expression matrices generated in this study have been deposited to the Gene Expression Omnibus (GEO) under the accession number GSE199669 and GSE228032. The reference genome of M. mulatta (Mmul_10) is available at https://hgdownload.soe.ucsc.edu/downloads.html. The published mouse data is downloaded from Gene Expression Omnibus (GSE172167). Source data are provided with this paper.

Human research participants

Policy information about studies involving human research participants and Sex and Gender in Research.

Reporting on sex and gender	No human participants were involved in this study.
Population characteristics	No human participants were involved in this study.
Recruitment	No human participants were involved in this study.
Ethics oversight	No human participants were involved in this study.

Note that full information on the approval of the study protocol must also be provided in the manuscript.

Field-specific reporting

Please select the one below that is the best fit for your research. If you are not sure, read the appropriate sections before making your selection.

☒ Life sciences ☐ Behavioural & social sciences ☐ Ecological, evolutionary & environmental sciences

For a reference copy of the document with all sections, see nature.com/documents/nr-reporting-summary-flat.pdf

Life sciences study design

All studies must disclose on these points even when the disclosure is negative.

Sample size	No statistical methods were used to predetermine sample size. Based on previous experience, experimental approach and published data, we used single-cell RNA-seq combined with single-nucleus RNA-seq, which was sufficient to obtain the number of cells necessary to perform a confident data analysis. The overall findings were also confirmed by immunohistochemistry, TEM analysis, lipid droplet staining and so on. A total of 12 adult female rhesus monkeys (Macaca mulatta) aged 4-7 years were used in this study. Two monkeys were used as control without any treatment, while the remaining ten animals were used to establish the spinal cord injury (SCI) model. Two animals for 7 days, 14 days, and 30 days each, four animals for 6 months and two of them were used for scaffold implantation. Among them, one animal was used for scRNA-seq/snRNA-seq and the other one was used for pathological analyses for each group.
Data exclusions	For single cell RNA-Seq data, we excluded data points through our quality control pipeline, as indicated in the methods section in the paper. In short, doublets were removed using DoubletFinder with the default parameters, and the 5% of cells most similar to the pseudo-doublets were excluded. We selected cells that met the following criteria: 1) greater than 500 detected genes; 2) UMI greater than 1000; 3) the percentage of mitochondrial counts smaller than 10; 4) the percentage of hemoglobin counts smaller than 5. 5) Genes that were expressed in more than 10 cells.
Replication	IHC assay were used throughout the manuscript to validated our findings. IHC, TUNEL and lipid droplets staining were replicated at least three replication attempts provided consistent results. scRNAseq were not replicated for the limitted animal number, but the sample size and number of analyzed cells were sufficiently powered.
Randomization	Animals were randomly assigned to each time points and groups for surgery and sequencing.
Blinding	The SCI experiments were not performed blinded for animal care requirements. For image acquisition, the region of interests were acquired on randomly selected tissue sections. Quantification of images was performed blinded.

Reporting for specific materials, systems and methods

We require information from authors about some types of materials, experimental systems and methods used in many studies. Here, indicate whether each material, system or method listed is relevant to your study. If you are not sure if a list item applies to your research, read the appropriate section before selecting a response.

Materials & experimental systems

n/a	Involved in the study
☐	☒ Antibodies
☒	☐ Eukaryotic cell lines
☒	☐ Palaeontology and archaeology
☐	☒ Animals and other organisms
☒	☐ Clinical data
☒	☐ Dual use research of concern

Methods

n/a	Involved in the study
☒	☐ ChIP-seq
☒	☐ Flow cytometry
☒	☐ MRI-based neuroimaging

Antibodies

Antibodies used

Primary antibodies:GFAP (GA5, Millipore, MAB360, 1:500), GFAP (abcam, ab4674,1:500), NeuN (EPR12763, abcam, ab177487, 1:500), NeuN (A60, Millipore, MAB377, 1:200), NPY (abcam, ab221145, 1:500), AIF1 (Wako, 019-19741, 1:500), AIF1 (abcam, ab5076, 1:500), IL-33 (EPR20417, abcam, ab207737, 1:200), SLC1A2 (abcam, ab41621, 1:200),Tuj-1 (Millipore, 05-559, 1:500), GPNMB (EPR22011-47, abcam, ab235873,1:400), VCAN (Invitrogen, MA5-34654, 1:200), GABA (Sigma, A2052,1:400), NF (Sigma, N4142, 1:400), MBP (ERP21188, abcam, ab218011, 1:500), APC (CC1, abcam, ab16794, 1:200), SERPINA3 (EPR14117(B), abcam, ab180492, 1:400), Ki67 (abcam, ab15580, 1:500), MPZ (Proteintech, 10572-1-AP, 1:500), SOX9 (EPR14335-78, abcam, ab185966, 1:500), and TNF-α (abcam,ab6671, 1:300).
Secondary antibodies: 488 Donkey anti-Mouse (Invitrogen, A21202, 1:500), 488 Donkey anti-Rabbit (Invitrogen, A21206, 1:500), 568 Donkey anti-Mouse (Invitrogen, A10037, 1:500), 568 Donkey anti-Rabbit (Invitrogen, A10042, 1:500), 647 Donkey anti-Chicken (Invitrogen, A78952, 1:500).

Validation

All antibodies used in this study were validated by the manufacturer or have been cited by other authors:
GFAP (GA5, Millipore, MAB360, 1:500)
https://www.merckmillipore.com/CN/zh/product/Anti-Glial-Fibrillary-Acidic-Protein-Antibody-clone-GA5,MM_NF-MAB360#
GFAP (abcam, ab4674,1:500)
https://www.abcam.com/products/primary-antibodies/gfap-antibody-ab4674.html
NeuN (EPR12763, abcam, ab177487, 1:500)
Spinal cord tissue engineering using human primary neural progenitor cells and astrocytes. DOI: 10.1002/btm2.10448
NeuN (A60, Millipore, MAB377, 1:200)
https://www.merckmillipore.com/CN/zh/product/Anti-NeuN-Antibody-clone-A60,MM_NF-MAB377
NPY (abcam, ab221145, 1:500)
https://www.abcam.com/products/primary-antibodies/neuropeptide-y-antibody-epr21877-ab221145.html
AIF1 (Wako, 019-19741, 1:500)
Lineage tracing reveals the origin of Nestin-positive cells are heterogeneous and rarely from ependymal cells after spinal cord injury.
https://doi.org/10.1007/s11427-020-1901-4
AIF1 (abcam, ab5076, 1:500)
https://www.abcam.com/products/primary-antibodies/iba1-antibody-ab5076.html
IL-33 (EPR20417, abcam, ab207737, 1:200)
https://www.abcam.com/il-33-antibody-epr20417-ab207737.html
SLC1A2 (abcam, ab41621, 1:200)
https://www.abcam.com/eaat2-antibody-ab41621.html
Tuj-1 (Millipore, 05-559, 1:500)
https://www.sigmaaldrich.cn/CN/zh/search/05-559?
focus=products&page=1&perpage=30&sort=relevance&term=05-559&type=product
GPNMB(EPR22011-47, abcam, ab235873,1:400)
https://www.abcam.com/gpnmb-antibody-epr22011-47-ab235873.html
VCAN(Invitrogen, MA5-34654, 1:200)
https://www.thermofisher.cn/cn/zh/antibody/product/Versican-Antibody-clone-JB42-32-Recombinant-Monoclonal/MA5-34654
GABA (Sigma, A2052,1:400)
https://www.sigmaaldrich.cn/CN/zh/product/sigma/a2052
NF(Sigma, N4142, 1:400)
https://www.sigmaaldrich.cn/CN/zh/product/sigma/n4142
Spinal cord tissue engineering using human primary neural progenitor cells and astrocytes. DOI: 10.1002/btm2.10448
MBP(ERP21188, abcam, ab218011,1:500)
https://www.abcam.com/myelin-basic-protein-antibody-epr21188-ab218011.html
APC(CC1, abcam, ab16794, 1:200)
https://www.abcam.com/apc-antibody-cc-1-ab16794.html
SERPINA3 (EPR14117(B), abcam, ab180492, 1:400)
https://www.abcam.com/products/primary-antibodies/aact-antibody-epr14117b-ab180492.html

	Ki67 (abcam, ab15580, 1:500)<br>https://www.abcam.com/products/primary-antibodies/ki67-antibody-ab15580.html<br>MPZ (Proteintech, 10572-1-AP, 1:500)<br>https://www.ptgcn.com/products/MPZ,P0-Antibody-10572-1-AP.htm<br>SOX9 (EPR14335-78, abcam, ab185966, 1:500)<br>https://www.abcam.com/products/primary-antibodies/sox9-antibody-epr14335-78-ab185966.html<br>TNF-α(abcam,ab6671, 1:300)<br>https://www.abcam.com/tnf-alpha-antibody-ab6671.html<br>In addition, a combination of morphological criteria, spatial distribution, and co-staining with other markers was used to validate each antibody used.

Animals and other research organisms

Policy information about studies involving animals; ARRIVE guidelines recommended for reporting animal research, and Sex and Gender in Research

Laboratory animals	Surgreries were conducted in adult female rhesus monkeys at the age of 4-7 years old. All animals were supplied and housed at Beijing Institute of Xieerxin Biology Resource with accreditation of Laboratory Animal Care accredited facility.
Wild animals	The study did not involve wild animals.
Reporting on sex	A total of 12 adult female monkeys were used in this study. No sex-based analyses were performed.
Field-collected samples	The study did not involve samples collected in the field.
Ethics oversight	All experimental procedures were in accordance with the Guide for the Care and Use of Laboratory Animals from the National Institutes of Health and approved by the Animal Care and Use Committee of Beijing Institute of Xieerxin Biology Resource (permit no. 20191017).

Note that full information on the approval of the study protocol must also be provided in the manuscript.
